# Immunological and clinical effects of probiotics

**DOI:** 10.1186/2045-7022-1-S1-S7

**Published:** 2011-08-12

**Authors:** Philippe Eigenmann

**Affiliations:** 1 Childen’s Hospital, Pediatric Allergy, Geneva, Switzerland

## 

An impact of the gut flora, the microbiota, on the modulation of allergies has been suspected for many years. Among non-pathogenic, colonizing organisms of the gut, probiotics play a special role in this regard and many experimental and clinical studies have focused on these micro-organisms. The presentation will first address immunologic concepts which are the basis of a potential action of probiotics, by presenting mostly experimental studies in animals. In the second part, clinical studies will be presented and will address mostly the timing of administration of probiotics, as well as specific effect on atopic disease prevention that have been studied. Finally, a critical review and interpretation based on the clinical studies available will be provided. The presentation should allow the attendance to understand which effect might be obtained by probiotics for prevention of atopic diseases. Selected references: 1 Sudo N, Sawamura S, Tanaka K, Aiba Y, Kubo C, Koga Y. The requirement of intestinal bacterial flora for the development of an ige production system fully susceptible to oral tolerance induction. J Immunol 1997; 159(4):1739-1745. 2 Majamaa H, Isolauri E. Probiotics: a novel approach in the management of food allergy. J Allergy Clin Immunol 1997; 99(2):179-185. 3 Kalliomaki M, Salminen S, Poussa T, Arvilommi H, Isolauri E. Probiotics and prevention of atopic disease: 4-year follow-up of a randomised placebo-controlled trial. Lancet 2003; 361(9372):1869-1871. 4 Viljanen M, Savilahti E, Haahtela T, Juntunen-Backman K, Korpela R, Poussa T et al. Probiotics in the treatment of atopic eczema/dermatitis syndrome in infants: a double-blind placebo-controlled trial. Allergy 2005; 60(4):494-500. 5 Kopp MV, Hennemuth I, Heinzmann A, Urbanek R. Randomized, Double-Blind, Placebo-Controlled Trial of Probiotics for Primary Prevention: No Clinical Effects of Lactobacillus GG Supplementation. Pediatrics 2008; 121(4):e850-e856. 6 Boyle RJ, Ismail IH, Kivivuori S, Licciardi PV, Robins-Browne RM, Mah LJ et al. Lactobacillus GG treatment during pregnancy for the prevention of eczema: a randomized controlled trial. Allergy 2010; doi: 10.1111/j.1398-9995.2010.02507.x.

